# Education and equipment for people who smoke crack cocaine in Canada: progress and limits

**DOI:** 10.1186/s12954-017-0144-3

**Published:** 2017-05-12

**Authors:** Carol Strike, Tara Marie Watson

**Affiliations:** 1 Dalla Lana School of Public Health, University of Toronto, 155 College Street, Toronto, Ontario M5T 3M7 Canada; 2 Centre for Addiction and Mental Health, Institute for Mental Health Policy Research, 33 Russell Street, Toronto, Ontario M5S 2S1 Canada

**Keywords:** Crack cocaine, Safer smoking, Needle and syringe program, Education, Best practice, Cross-sectional, Canada

## Abstract

**Background:**

People who smoke crack cocaine experience a wide variety of health-related issues. However, public health programming designed for this population is limited, particularly in comparison with programming for people who inject drugs. Canadian best practice recommendations encourage needle and syringe programs (NSPs) to provide education about safer crack cocaine smoking practices, distribute safer smoking equipment, and provide options for safer disposal of used equipment.

**Methods:**

We conducted an online survey of NSP managers across Canada to estimate the proportions of NSPs that provide education and distribute safer smoking equipment to people who smoke crack cocaine. We also assessed change in pipe distribution practices between 2008 and 2015 in the province of Ontario.

**Results:**

Analysis of data from 80 programs showed that the majority (0.76) provided education to clients on reducing risks associated with sharing crack cocaine smoking equipment and about when to replace smoking equipment (0.78). The majority (0.64) also distributed safer crack cocaine smoking equipment and over half of these programs (0.55) had done so for less than 5 years. Among programs that distributed pipes, 0.92 distributed the recommended heat-resistant Pyrex and/or borosilicate glass pipes. Only 0.50 of our full sample reported that their program provides clients with containers for safer disposal of used smoking equipment. The most common reasons for not distributing safer smoking equipment were not enough funding (0.32) and lack of client demand (0.25). Ontario-specific sub-analyses showed a significant increase in the proportion of programs distributing pipes in Ontario from 0.15 (2008) to 0.71 (2015).

**Conclusions:**

Our findings point to important efforts by Canadian NSPs to reduce harm among people who smoke crack cocaine through provision of education and equipment, but there are still limits that could be addressed. Our study can provide guidance for future cross-jurisdiction studies to describe relationships involving harm reduction programs and provision of safer crack cocaine smoking education and equipment.

**Electronic supplementary material:**

The online version of this article (doi:10.1186/s12954-017-0144-3) contains supplementary material, which is available to authorized users.

## Background

Although the harm reduction philosophy that has moved forward in Canada, and North America more broadly, is inclusive of people who consume a wide spectrum of psychoactive substances, actual programming has been more focused on people who inject drugs. This is concerning from a public health perspective because in Canada crack cocaine use is common among street-based people who use drugs [1–3]. People who smoke crack cocaine report experiencing oral sores, cuts, and burns that are connected to the use of improvised crack pipes fashioned out of hazardous glass and metal materials [4, 5], and such injuries may facilitate infectious disease transmission when pipes are shared among users [6, 7]. Pipe sharing is also commonly reported, especially when pipes are difficult to obtain [8–10]. Indeed, evidence shows elevated rates of hepatitis C virus (HCV), as well as HIV and other infectious diseases, among people who smoke crack cocaine [11–15].

**Fig. 1:**
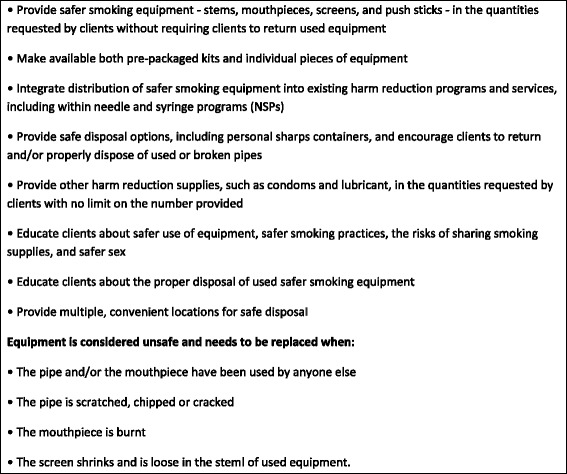
Recommended best practice policies to facilitate smoking with a pipe – stem, mouthpiece, and screen – which is made from materials that are non-hazardous to health and have never been shared

There are likely various, and some convergent, reasons why harm reduction programming for people who smoke crack cocaine has lagged behind programming developed for people who inject drugs. Injection drug use has long been considered the riskiest form of drug use in terms of potential health-related risks and as such public health authorities have prioritized services, especially HIV prevention services, for people who inject drugs (e.g., [16]). Nonetheless, although people who use illicit drugs in general are a socially marginalized group, people who smoke crack cocaine often exhibit pronounced marginalization characterized by, for example, poverty, unstable housing or homelessness, and elevated rates of encounters with the criminal justice system (e.g., [1, 17–19]). The establishment of greater services for this drug-using population has relied on additional advocacy efforts. Harm reduction advocates in Toronto and Vancouver were among the first groups in Canada to recommend and begin distribution of safer smoking equipment to engage people who smoke crack cocaine in programming [20, 21]. However, implementation of policies and interventions designed for crack cocaine users has also been hindered and delayed by questions about the legality of the distribution of safer smoking equipment and related opposition from police (cf. [19, 22–26]). In an effort to promote programming that addresses high rates of HCV among people who smoke crack cocaine, Canadian best practice recommendations encourage needle and syringe programs (NSPs) and other harm reduction programs to provide education on safer crack cocaine smoking practices and use of smoking equipment; distribute safer smoking equipment (i.e., Pyrex and/or borosilicate glass pipes or “stems”, mouthpieces, screens, and push sticks); and provide options for safer disposal of used equipment [27]. See Fig. 1 for a snapshot of the complete set of these best practices pertaining to safer crack cocaine use. These evidence-based guidelines for safer crack cocaine smoking education and equipment distribution were developed by a national, multi-stakeholder team (for a description of the best practices team formation, composition, and collaboration, see [28]).

As part of a national-in-scope evaluation of NSP practices and policies, the first of its kind that we are aware of in Canada, we conducted a survey of program managers to estimate the proportions of NSPs providing education and distributing safer smoking equipment to people who smoke crack cocaine. For the province of Ontario, we used previously collected survey data [25] to assess if there had been a change in the distribution of safer smoking equipment over time.

## Methods

Managers of NSPs across Canada were invited to participate in an online survey examining program policies and uptake of best practices. Eligible programs included those operated by a public health organization or other agency contracted by their local health unit to provide needle/syringe distribution in any province or territory. We focused on these “core” programs associated with public health units and did not attempt to sample “satellite” NSP services (see [25]). To expand their reach, core NSPs often engage other organizations to be satellite sites that can also offer NSP services. Trying to sample *all* NSPs including satellite sites would have been a time-consuming effort and one that might not have added much value as core NSPs often provide their local satellite services with the necessary training (including policies and procedures to follow), supplies, and support (see again [25]). As there is no central registry of all NSPs in Canada, we created an email address list using three approaches. First, we knew from best practices research team members that three provinces (Quebec, Ontario, and British Columbia) kept their own comprehensive and up-to-date lists of all NSPs (including program manager email addresses) for their respective regions. We obtained these lists for Quebec and Ontario. An official from the western province of British Columbia opted out of providing their email list stating that the burden of participation was too great for local NSP managers who were at that time implementing new overdose prevention programming. We did not have the time and resources necessary to contact all public health units in British Columbia and then follow up with all NSPs in that province to obtain the requisite email addresses. For the remaining provinces where, in some cases, there was a small number of programs and local managers knew each other, we asked the regional representative on the best practices team to provide email contact information for NSP managers in their province. Lastly, for the territories, the first author contacted local harm reduction representatives and a territorial ministry of health to identify NSP managers in those regions. The northernmost territory, Nunavut, did not have an NSP. Using these three approaches, we believe that we captured the email addresses for managers of all operational core NSPs in Canada, with the exception of NSPs in British Columbia.

To encourage survey participation, we modified a method by Dillman et al. [29] by asking team members who were involved in harm reduction policy and/or service provision in their regions to send to their local NSP managers an initial email “alert” to introduce the study and advise of upcoming invitations to participate. One to 2 weeks after these alerts were sent, the first author sent formal email invitations to potential participants in each province and territory; these invitations included a study information sheet with consent form and a link to the survey. Two weeks after these invitations, potential participants were sent the first email reminder about the survey. Two weeks after these reminders, we emailed potential participants a final reminder about completing the survey. To provide incentive to participate in the online survey, we offered to all potential participants an option to enter a draw to win for their program one of 20 gift cards worth $100CAD for a well-known coffee shop chain. Study recruitment was staggered and the survey was open to participants from April 9 to August 4, 2015.

Participants were asked questions (in Yes/No, multiple choice, Likert scale, and open-ended formats) about their program characteristics, distribution of harm reduction materials, including safer crack cocaine smoking equipment, and other key topics identified in the best practice recommendations [27, 30]. The questionnaire was developed for an online platform, FluidSurveys, and was offered in English and French. Please see Additional file 1 that contains English online survey text that is relevant to the findings we report in this article. Before launching data collection, we pilot tested the online survey with five program managers from different provinces and modified some questions as per their feedback. The University of Toronto Research Ethics Board (REB) approved this study.

Data were downloaded, managed, and analyzed using SPSS (version 24). Specifically, we report frequency distributions and bivariate statistics to characterize the proportion of programs offering safer crack cocaine smoking education and equipment distribution by NSPs. In addition, using data from an earlier study that used the same online survey methods for Ontario [25], we compared the proportion of programs in that province that distributed pipes in 2008 versus 2015. Similar data were not available for the other provinces or territories.

## Results

### Sample characteristics

We invited 125 NSP managers from across Canada to complete the online survey. A filter question identified eight managers who were not eligible to participate because their program did not actively distribute needles at that time (our only study eligibility criterion). Of the remaining 117 potential participants, 104 initially responded to the survey; upon reviewing the data, we removed 24 surveys because of incomplete data, leaving 80 surveys for these analyses. Table 1 presents program characteristics. Throughout our results, we report the proportion of programs reporting each characteristic or practice.

**Table 1:** NSP characteristics as reported by program managers

Program characteristic	Proportion of programs reporting characteristic (*n*)
NSP located within a	
•Public health unit	0.40 (32)
•Community-based organization	0.36 (29)
•AIDS service organization	0.20 (16)
•Community health center	0.04 (3)
Region	
•Ontario	0.36 (28)
•Quebec	0.32 (25)
•Western and Northern (includes Manitoba, Saskatchewan, Alberta, Northwest Territories, and Yukon)	0.19 (15)
•Atlantic (includes New Brunswick, Nova Scotia, Prince Edward Island, and Newfoundland and Labrador)	0.13 (10)
Annual program budget (in CAD)	
•Between $50,000 and $250,000	0.35 (26)
•Over $250,000	0.26 (19)
•Less than $50,000	0.23 (17)
•No annual budget for NSP	0.16 (12)
Volume of needles distributed annually	
•Over 100,000	0.49 (39)
•Between 10,000 and 99,999	0.26 (21)
•Less than 10,000	0.25 (20)
In operation for 10 years or longer	0.81 (62)

### Provision of safer crack cocaine smoking education

A majority of participants (0.76) reported that their program provides education to clients on reducing risks associated with sharing crack cocaine smoking equipment. Further, 0.75 indicated that their program provides education on identifying risks, such as cuts and injuries, from the use of improvised smoking equipment (e.g., soda cans as makeshift pipes), and 0.72 reported that they provide education on how to use safer smoking equipment.

Over three quarters of participants (0.78) reported that their program staff advise clients about when to replace smoking equipment. In terms of specific instances when it is time to replace smoking equipment, 0.75 of managers reported that their program advises clients to replace pipes and/or mouthpieces if these items have been used by anyone else; 0.74 advise clients to replace their pipe if it is scratched, chipped, or cracked; 0.71 advise clients to replace mouthpieces that are burnt; and 0.70 advise clients to replace the screen if it shrinks and becomes loose in the pipe.

We asked participants about the formats their programs use to provide education to clients about drug-related risk behaviors and practices. Given the general way in which we framed these questions, we cannot determine if and where the responses pertain to delivery of education on injection- or smoking-related behaviors, or both. With that caveat, we restricted these analyses to only managers who reported that their program provides education regarding how to use safer smoking equipment (*n* = 58) and found that all reported that their program offers educational information pamphlets or brochures; 0.97 offer one-on-one counseling; 0.79 offer demonstrations; 0.52 offer peer-delivered education; 0.38 offer skills-building sessions or group education; and 0.09 use instructional videos.

### Distribution of safer crack cocaine smoking equipment

When asked if their program distributes “any” safer crack cocaine smoking equipment, 0.64 of managers responded affirmatively. Of these participants, nearly all (0.96) indicated that their program distributes pipes; over half (0.55) reported that their program has distributed safer smoking equipment for less than 5 years, while 0.43 have done so for more than 5 years and the remainder did not know how long their program has distributed this equipment. Of the programs that distribute pipes, 0.92 reportedly distribute the recommended heat-resistant Pyrex and/or borosilicate glass pipes, while 0.08 distribute pipes of an unknown type of glass. The proportions of managers who reported distribution of other pieces of safer smoking equipment were as follows: 0.94 for mouthpieces, 0.94 for screens, and 0.92 for push sticks. In addition to offering each piece of recommended equipment separately, 0.86 indicated that their program offers pre-packaged kits containing pipes plus other safer smoking equipment. In short, most of the programs that distribute safer smoking equipment reported giving out the recommended types of pipes as well as a complement of other safer smoking materials. Only 0.50 of our full sample reported that their program provides clients with containers for safer disposal of used smoking equipment.

Among participants who reported that their program does not distribute safer smoking equipment (0.35), the two most commonly endorsed reasons for not doing so were not enough funding (0.32) and lack of client demand (0.25). Only two participants selected “opposition from law enforcement” as a reason. Six managers wrote additional reasons in their surveys and three of these indicated that their programs are seeking to implement safer crack cocaine smoking equipment distribution and/or have received recent approval to do so.

When asked about distribution policies, 0.53 of managers who indicated that their program distributes pipes reported no maximum on the number of pipes that they will provide to a client at any one time; the remaining 0.47 indicated that their program sets a maximum. Reported limits ranged from one to 20 pipes per visit, though most commonly participants (0.57) indicated that their program imposes a maximum of one or two pipes per client at a time. When asked why their program imposes limits on pipe distribution, 0.61 of these managers reported that this quantity adequately meets client demand and 0.52 reported concerns about running out of supplies. Several participants added more information to their surveys that suggested that maximums are imposed due to concerns about clients selling their pipes on the street (e.g., “Some clients have been known to sell what they don’t use”). One participant added that a benefit of having a pipe limit is that it keeps clients who smoke crack cocaine coming back to their program for services (i.e., “to maintain continuity of contact with the clients so that we can provide support, education, and referrals”).

### Influence of best practices on safer smoking education and distribution practices

Also as part of the online survey, we asked managers if they and their staff had used the recent set of national best practice recommendations [27] to change and align program practices with said evidence-based guidance. Just under half of participants (0.49) reported that their program used the recommendations to influence pipe distribution practices, 0.49 also did so to influence safer smoking education practices, and 0.39 did so to influence other smoking equipment (e.g., mouthpieces, screens) distribution practices.

### Ontario: more programs distributing pipes over time

Finally, to examine potential changes in pipe distribution over time, we performed Ontario-specific sub-analyses and compared evaluation data collected in 2008 [25] with data from the 2015 survey. Analysis showed a significant increase in the proportion of programs distributing pipes in Ontario from 0.15 to 0.71.

## Discussion

Our findings show that many NSPs in Canada offer safer crack cocaine smoking education and distribute equipment as recommended. In Ontario, there has been a significant increase in the number of programs distributing crack cocaine pipes since 2008. These findings are encouraging given that crack cocaine smoking occurs in cities across Canada and increases in use have been documented in some locations (e.g., [1–3]), and elevated rates of HCV and other health-related problems are reported among people who smoke crack cocaine [11–15], major reasons to engage this population in harm reduction services. However, there are important questions our study leaves unanswered or open for future investigations.

Although we found that some programs reported using the national best practice recommendations to influence safer crack cocaine education- and equipment-related practices, due to the cross-sectional nature of our survey and how we worded specific questions, we cannot state whether or not dissemination of said recommendations led to the observed levels of education provision and any increases in equipment distribution by NSPs. Overall, the evidence base supporting services for people who smoke crack cocaine is evolving and not as developed as that which supports harm reduction services for people who inject drugs—the latter backed by decades of research and recommended by international associations such as the World Health Organization [31]. More research is needed, for example, to solidify the linkage(s) between sharing pipes and HCV transmission [6]. That said, recent evidence demonstrates that access to safer smoking equipment can help to reduce health-related harm [32]. Research evidence is, nonetheless, one among numerous factors that impact health-related policy decisions.

Our study indicates an ongoing need to investigate and address barriers to best practice uptake, as 35% of managers in our sample reported that their program does not distribute any safer crack cocaine smoking equipment. More work is needed to address other domains found to promote uptake of evidence-based recommendations, including nurturing champions of organizational change, organizational cultures that support innovation and leaderships that promote the use of evidence-based practice, and ensuring adequate funding streams for distribution and disposal of safer smoking equipment [33, 34]. Only two managers among those who said that their programs do not distribute pipes selected police opposition as an underlying reason. This finding seems consistent with results from our larger evaluation study which show that the majority of NSP managers we sampled reported mostly positive relationships with their local law enforcement [35]. However, interpretation of this finding is difficult in light of other research that has reported policing practices to be a barrier to services designed for people who smoke crack cocaine (e.g., [19, 24]). Police support and opposition regarding harm reduction programs are dynamic, though, for example, in Canada there are signs that police perspectives on supervised injection facilities have changed in recent years, seemingly linked to the opioid overdose epidemic (cf. [36, 37]). How police may view services for people who smoke crack cocaine and how those views are changing or may change are worthy of in-depth investigation.

Lastly, although collection and safer disposal of used injection equipment is a core activity of NSPs, including providing clients with rigid, tamper-resistant, and clearly labeled sharps containers (see [27] for evidence-based best practice recommendations regarding disposal and handling of used drug-use equipment), we found that only half of all NSPs that we sampled provide clients with containers for safer disposal of used smoking equipment. We did not include more detailed or follow-up questions about this issue in the online survey, so we are unsure if this lack of safer disposal container provision represents a resource or cost issue and/or something else. We know from anecdotal reports from members of the cross-regional, multi-stakeholder best practice team that cost can be a barrier and some programs already struggle to cover the costs of injection equipment disposal. It is also possible that NSP staff do not regard pipes and other safer smoking equipment as sharps and/or biohazard material requiring the same level of safety procedures as used injection equipment. The removal from circulation and safer disposal of used injection equipment have long been considered key elements of NSP strategies to reduce needle reuse and accidental needle-stick injuries which, in turn, reduce opportunities for infectious disease transmission [38, 39]. More research is needed to determine if disposal is similarly as important for reducing certain risks associated with crack cocaine smoking.

In terms of study limitations, our findings may not be generalizable across all programs in Canada. One province with many NSPs and other harm reduction programs did not participate. It is also possible, though perhaps unlikely, that there are some programs that distribute safer smoking equipment and no injection equipment, and these would have been excluded from our survey. Although not an ideal sample, we otherwise captured data from programs from all other regions, including the Maritimes and the northern territories, and are thus able to provide a highly unique snapshot of Canadian practices. Our findings can provide some guidance for future, larger-sample investigations to describe and report on relationships involving harm reduction programs and safer crack cocaine smoking education and equipment.

## Conclusions

Our findings point to important efforts on the part of Canadian NSPs to help reduce HCV and other health-related harm among people who smoke crack cocaine through provision of education and equipment that aim to address such harm. HCV is a preventable infection, and although at times challenging to implement harm reduction interventions, increased efforts are needed to reduce drug-related HCV risk in Canada and elsewhere in the world. Although beyond the scope of this article, we also stress that while client education and equipment distribution have a role to play in reducing such risk, ultimately improving the health and well-being of people who smoke crack cocaine requires much broader attention to social-structural factors—including social marginalization and drug law enforcement—that continue to disproportionately impact this population and drive much of their drug-related risk behaviors [40].

## Additional file

Additional file 1: Best Practice Recommendations Survey. (DOC 70 kb)

## Abbreviations

HCV: Hepatitis C virus
HIV: Human immunodeficiency virus
NSPs: Needle and syringe programs
