# Supplementary material for: Education and equipment for people who smoke crack cocaine in Canada: progress and limits
Source: Harm Reduct J. 2017 May 12;14:17. doi: 10.1186/s12954-017-0144-3 (PMC5427547; doi:10.1186/s12954-017-0144-3)
Supplement: Additional file 1: — Best Practice Recommendations Survey. (DOC 70 kb) [file 12954_2017_144_MOESM1_ESM.doc]

# *Best Practice Recommendations* Survey

*(Note to readers: Below is a modified and unformatted version of the questionnaire that was formatted and administered using FluidSurveys.)*

***Preamble***

In this survey, you will be asked questions relating to your needle and syringe program (NSP)/harm reduction program’s policies, practices, and basic statistics. We are not asking about the entire Public Health Unit or Community Health Centre that the program may be a part of, if applicable. We are, however, interested in the full NSP/harm reduction program that provides services to people who use drugs.

We recognize that programs vary in terms of budgets and other factors that may constrain their practices. Please answer each question to the best of your knowledge. You are able to skip any question(s) that you do not feel like answering. The survey may take between 20 to 30 minutes to complete. You have the option to save and return to your survey at a later time.

**Section AA: NSP/Harm Reduction Program Information**

AA1 Does your program distribute needles and syringes? Yes *(1)* or No *(0)*

*If No, skip to the termination*

AA2 Is your program part of a:

- *(1)* Public Health Unit
- *(2)* Community Health Centre
- *(3)* AIDS Service Organization
- *(4)* Community-Based Service Organization

AA3 Is your program located on-reserve? Yes *(1)* or No *(0)*

AA4 In what province/territory is your program located?

- Alberta
- Manitoba
- New Brunswick
- Newfoundland and Labrador
- Nova Scotia
- Ontario
- Prince Edward Island
- Quebec
- Saskatchewan
- Northwest Territories
- Nunavut
- Yukon

AA5 How many years has your NSP/harm reduction program been operating? ________

AA8 What is the annual budget for your program?

1. No budget for the NSP/harm reduction program
2. < $50,000
3. $50,000 – $99,999
4. $100,000 – $250,000
5. > $250,000

# Section B: Safer Crack Cocaine Smoking Equipment Distribution

*B1_1.* Does your NSP/harm reduction program advise clients that smoking equipment should be replaced? Yes *(1)* or No *(0)*

If no, skip to B2

B1_2. Does your NSP/harm reduction program advise clients that smoking equipment should be replaced when: Please answer 'Yes' or 'No' to ALL.

B1a – the pipe and/or the mouthpiece have been used by anyone else

Yes (1) or No (0)

B1b – the pipe is scratched, chipped, or cracked Yes (1) or No (0)

B1c – the mouthpiece is burnt Yes (1) or No (0)

B1d – the screen shrinks and is loose in the stem Yes (1) or No (0)

B1e – do not know Yes (1) or No (0)

B2. Does your NSP/harm reduction program provide clients with containers for disposal of used safer smoking equipment? Yes *(1)* or No *(0)*

B3. Does your NSP/harm reduction program distribute any safer crack cocaine smoking equipment? Yes *(1)* or No *(0)*

*If Yes, skip to B5*

B4. Why does your NSP/harm reduction program not distribute safer crack cocaine smoking equipment? Please answer 'Yes' or 'No' to ALL. (if answering this question, skip to C1 next section)

B4a – decision by NSP/harm reduction program manager Yes (1) or No (0)

B4b – decision by other health-related bodies (e.g., Ministry of Health, Board of

Health) Yes (1) or No (0)

B4c – decision by municipal body/city council Yes (1) or No (0)

B4d – opposition from law enforcement agents Yes (1) or No (0)

B4e – not enough funding for these supplies Yes (1) or No (0)

B4f – our clients do not want safer smoking equipment/no demand

Yes (1) or No (0)

B4g – do not know Yes (1) or No (0)

B4h – other reasons (please specify) ____________________

B5. How long has your NSP/harm reduction program been distributing safer crack cocaine smoking equipment?

1. less than 5 years
2. more than 5 years

*(9)* do not know

B6. Does your NSP/harm reduction program distribute stems?

B6a – yes, heat-resistant Pyrex and/or borosilicate glass stems *(1)*

B6b – yes, but stems of an unknown type of glass *(2)*

B6c – no, the program does not distribute stems *(0)*

*If no, skip to B10*

B7. Does your NSP/harm reduction program offer pre-packaged safer crack cocaine smoking kits containing stems and other safer smoking equipment? Yes *(1)* or No *(0)*

B8. What is the maximum number of stems your NSP/harm reduction program will give to a client at any one time?

1. enter maximum  *________*

*(0)* no maximum number *(skip to B10)*

B9. Does your NSP/harm reduction program place limits or a maximum on the number of stems that clients can obtain at any one time because of:

Please answer 'Yes' or 'No' to ALL.

B9a – not enough funding to distribute more Yes *(1)* or No *(0)*

B9b – the policy is adequate for meeting client demand Yes *(1)* or No *(0)*

B9c – decision by NSP/harm reduction program manager, Executive Director,

and/or Ministry of Health Yes *(1)* or No *(0)*

B9d – concerns about running out of supplies or shortages Yes *(1)* or No *(0)*

B9e – do not know Yes *(1)* or No *(0)*

B9f – other reason(s) (please specify) ________________________

B10. Does your NSP/harm reduction program distribute the following? Please answer 'Yes' or 'No' to ALL.

B10a – mouthpieces Yes *(1)* or No *(0)*

B10b – push sticks Yes *(1)* or No *(0)*

B10c – screens Yes *(1)* or No *(0)*

B10d – pre-packaged safer crack cocaine smoking kits containing other safer smoking equipment only (without stems) Yes *(1)* or No *(0)*

B10e – do not know Yes *(1)* or No *(0)*

**Section C: Safer Drug Use Education**

C2. Does your NSP/harm reduction program provide education to help clients who smoke drugs like crack cocaine. Please answer 'Yes' or 'No' to ALL.

C2a – know how to use safer smoking equipment Yes (1), No (0), Not Applicable (9)

C2b– reduce risks of sharing smoking supplies Yes (1), No (0), Not Applicable (9)

C2c – identify risks, such as cuts and injuries, from the use of improvised smoking supplies (e.g., soda cans, Brillo) Yes (1), No (0), Not

Applicable (9)

C2d – other (please specify) _____________________

C3. In what format does your NSP/harm reduction program provide education to clients about risk behaviours and practices? Please answer 'Yes' or 'No' to ALL.

C3a – one-on-one education Yes (1) or No (0)

C3b – skills-building sessions or group education Yes (1) or No (0)

C3c – information pamphlets or brochures Yes (1) or No (0)

C3d – instructional videos Yes (1) or No (0)

C3e – demonstrations Yes (1) or No (0)

C3f – peer-delivered interventions Yes (1) or No (0)

C3g – other (please specify) ________________________

# Section E: Use of the *Best Practice Recommendations for Canadian Harm Reduction Programs that Provide Service to People Who Use Drugs and are at Risk for HIV, HCV, and Other Harms: Part 1* (BPR Part 1)

E7. Has your NSP/harm reduction program used BPR Part 1 to make changes to program practices and policies regarding the following areas to bring them more in line with recommended practices? Please answer 'Yes' or 'No' to ALL.

E7a – needle distribution Yes *(1)* or No *(0)*

E7b – cooker distribution Yes *(1)* or No *(0)*

E7c – other injection equipment distribution (e.g., filter, acidifiers) Yes *(1)* or No *(0)*

E7d – disposal of used needles and injection equipment Yes *(1)* or No *(0)*

E7e – distribution of stems for safer crack cocaine smoking Yes *(1)* or No *(0)*

E7f – other safer crack cocaine smoking equipment (e.g., mouthpieces, screens) Yes *(1)* or No *(0)*

E7g – safer injection education Yes *(1)* or No *(0)*

E7h – safer crack cocaine smoking education Yes *(1)* or No *(0)*

E7i – general education and training on overdose prevention Yes *(1)* or No *(0)*

E7j – naloxone distribution Yes *(1)* or No *(0)*

E7k – other (please specify) ______________________
